# Neuroprotection by Abdominal Ultrasound in Lipopolysaccharide-Induced Systemic Inflammation

**DOI:** 10.3390/ijms24119329

**Published:** 2023-05-26

**Authors:** Wen-Shin Song, Tai-Ho Hung, Shing-Hwa Liu, Yin-Ting Zheng, Hsin-Mei Lin, Feng-Yi Yang

**Affiliations:** 1 Division of Neurosurgery, Cheng Hsin General Hospital, Taipei 112, Taiwan; 2 Department of Neurological Surgery, Tri-Service General Hospital, National Defense Medical Center, Taipei 114, Taiwan; 3 Department of Obstetrics and Gynecology, Taipei Chang Gung Memorial Hospital, Taipei 105, Taiwan; 4 Department of Obstetrics and Gynecology, Keelung Chang Gung Memorial Hospital, Keelung 204, Taiwan; 5 Institute of Toxicology, College of Medicine, National Taiwan University, Taipei 106, Taiwan; 6 Department of Medical Research, China Medical University Hospital, China Medical University, Taichung 404, Taiwan; 7 Department of Biomedical Imaging and Radiological Sciences, National Yang Ming Chiao Tung University, Taipei 112, Taiwan

**Keywords:** colon, inflammation, neuroinflammation, apoptosis, microglia, gut–brain axis

## Abstract

Systemic inflammation is associated with intestinal inflammation and neuroinflammation by imbalancing the gut–brain axis. Low-intensity pulsed ultrasound (LIPUS) has neuroprotective and anti-inflammatory effects. This study explored LIPUS’s neuroprotective effects against lipopolysaccharide (LPS)-induced neuroinflammation through transabdominal stimulation. Male C57BL/6J mice were intraperitoneally injected with LPS (0.75 mg/kg) daily for seven days, and abdominal LIPUS was applied to the abdominal area for 15 min/day during the last six days. One day after the last LIPUS treatment, biological samples were collected for microscopic and immunohistochemical analysis. Histological examination showed that LPS administration leads to tissue damage in the colon and brain. Transabdominal LIPUS stimulation attenuated colonic damage, reducing histological score, colonic muscle thickness, and villi shortening. Furthermore, abdominal LIPUS reduced hippocampal microglial activation (labeled by ionized calcium-binding adaptor molecule-1 [Iba-1]) and neuronal cell loss (labeled by microtubule-associated protein 2 [MAP2]). Moreover, abdominal LIPUS attenuated the number of apoptotic cells in the hippocampus and cortex. Altogether, our results indicate that abdominal LIPUS stimulation attenuates LPS-induced colonic inflammation and neuroinflammation. These findings provide new insights into the treatment strategy for neuroinflammation-related brain disorders and may facilitate method development through the gut–brain axis pathway.

## 1. Introduction

Inflammatory bowel disease (IBD) mainly comprised of ulcerative colitis and Crohn’s disease is characterized by chronic inflammation of the gastrointestinal tract. The incidence and prevalence of IBD have increased rapidly over recent decades, especially in Europe and North America [1]. Increasing evidence indicates that IBD is associated with neuroinflammation and neurodegenerative diseases, including Alzheimer’s and Parkinson’s disease [2,3]. While biological treatments have recently been developed, there is no effective cure for IBD [4]. Recent studies have reported communication between the gut and neurological diseases through the gut–brain axis [5,6]. In addition, systemic inflammation will lead to an imbalance in the gut–brain axis. Indeed, systemic inflammation can induce proinflammatory cytokines and stimulate microglia in the brain [7,8]. Therefore, the gut–brain axis signaling pathways have been implicated in systemic inflammation and neuroinflammation [9].

Microglia, the immune cells in the brain, play crucial roles in neuroinflammation [10]. Activated microglia have beneficial or harmful roles in the brain depending on the duration of the inflammatory response [11]. Chronic microglial activation leads to an increase in proinflammatory mediators and neuronal death. Therefore, attenuating microglial activation is a therapeutic strategy in treating neuroinflammation-related brain diseases [12]. In addition, microtubule-associated protein 2 (MAP2), a marker of dendrisomatic neuronal injury, is a structural protein maintaining neuroarchitecture. Neuronal death is accompanied by MAP2 loss. A reduction in MAP2 may be an initial neuronal dysfunction sign and be involved in neurodegenerative and neuropsychiatric disorders [13]. Deficient MAP2 immunoreactivity in postmortem brains is a hallmark of schizophrenia, and increasing MAP2 levels is a potential therapeutic approach for treating schizophrenia [14,15].

There are several kinds of animal models of colonic inflammation to study IBD. Administration of lipopolysaccharide (LPS), a cell-wall component of gram-negative bacteria, is commonly used to induce a systemic inflammatory response in animal models. When the levels of LPS present in the gut are sufficiently high, they may increase the permeability of the gut, thereby causing weakness of the gut to pathogenic bacteria [16]. The increased permeability of the gut barrier can result in increased passage of endotoxins from inside the gut to the systemic circulation. The recognition of LPS by toll-like receptor 4 (TLR4) then stimulates the activation of the nuclear factor kappa B (NF-κB) signaling pathway and induces the expression of proinflammatory mediators, such as TNF-α, IL-1β, and IL-6. The intestinal barrier separates the intestinal tract from the inner host and prevents toxic substances from entering the body [17]. Disruption of intestinal barrier function is a central feature of IBD [18]. LPS in gut microbiota increases blood LPS levels via intestinal inflammation. LPS administration leads to systemic inflammation and neuroinflammation in the brain [19]. LPS impairs intestinal barrier function and induces inflammation. Intestinal barrier dysfunction underlies many diseases, including IBD and neurodegenerative diseases. Therefore, reversing inflammation-induced barrier impairment could be a potential therapeutic approach for IBD and neuroinflammation-related diseases.

In recent years, numerous studies reported that intestinal microbiota can affect the central nervous system (CNS) via the gut–brain axis. The microbiota–gut–brain axis is important for the maintenance of brain homeostasis through neural, immune, metabolic, and endocrine pathways. Alterations in the composition of the intestinal microbiota result in brain disorders by contributing to the impairment of microglia function, mostly due to LPS-induced oxidative stress [20]. Moreover, chronic intestinal inflammation may lead to the development of neurodegenerative diseases, such as Alzheimer’s disease and Parkinson’s disease. Therefore, a strategic breakthrough in the treatment of neurodegenerative diseases may result from remodeling and restoring gut microbiota composition [21].

It has been shown that low-intensity pulsed ultrasound (LIPUS) stimulation attenuated TLR4/NF-κB-induced neuroinflammation and inhibited proinflammatory cytokine release in vivo and in vitro [22,23,24]. Our previous results provide a better understanding of how LIPUS stimulation regulates anti-inflammatory actions in microglia, providing further evidence that such stimulation may be regarded as a novel strategy for the treatment of neuroinflammation. Recent studies reported that ultrasound could improve IBD symptoms via abdominal stimulation [25]. Ultrasound-attenuated dextran sulfate sodium (DSS) induced colitis via beneficial anti-inflammation effects and decreased colonic epithelial barrier permeability [26]. However, whether abdominal ultrasound stimulation can ameliorate neuroinflammation-induced brain injury by dampening intestinal barrier dysfunction remains unknown. This study induced a mouse model of systemic inflammation by LPS injection. It then explored the effects of LIPUS treatment on LPS-induced brain injury and colonic barrier deficits in mice through its effects on barrier function in the colon, brain structure, and neuronal death.

## 2. Results

### 2.1. Effect of LIPUS on Spleen Weight in LPS-Treated Mice

**Figure 1:**
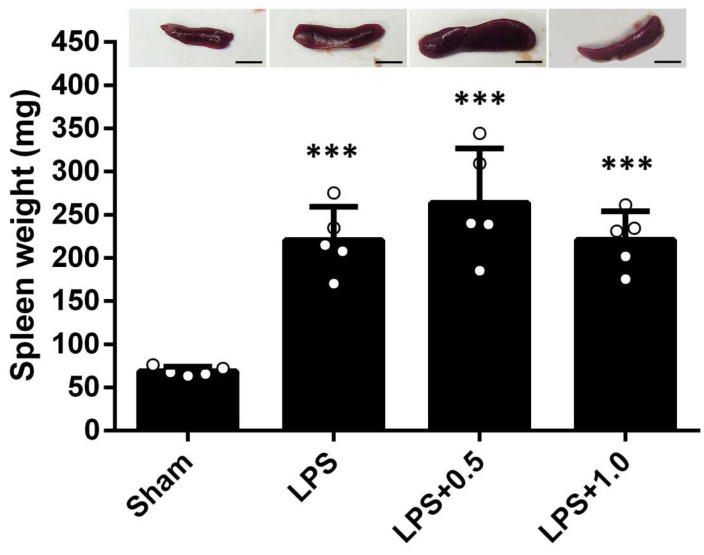
Effect of LIPUS treatment on spleen weight in LPS-induced systemic inflammation. LPS increased spleen weight. LIPUS treatment did not affect these values. Scale bar = 5 mm. Key: ***, *p* < 0.001 compared to the sham group (*n* = 5). Each circle indicates one sample of group.

The spleen is the largest peripheral lymphatic organ and plays a crucial role in the inflammatory response. The degree of inflammation usually correlates with the increased spleen weights. To evaluate the association between the spleen and systemic inflammation, we investigated the change in spleen weight after LPS administration. Spleen weights were significantly higher in the LPS groups than in the Sham group (220.86 ± 38.52 vs. 69.00 ± 5.20, *p* < 0.001; Figure 1), indicating that LPS strongly affects spleen water content in endotoxic mice. However, spleen weights were similar in the LPS+LIPUS 0.5, LPS+LIPUS 1.0, and LPS groups. Therefore, LIPUS treatment did not alleviate LPS-induced spleen edema.

### 2.2. Effects of LIPUS on Histological Changes in the Colon of LPS-Treated Mice

**Figure 2:**
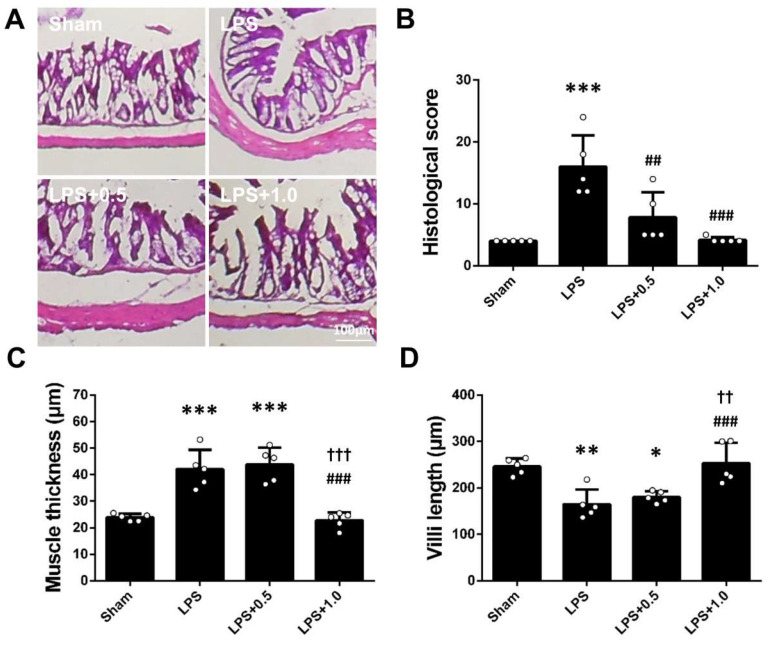
Colon changes in LPS-treated mice after LIPUS treatment. (**A**) Representative images of H&E staining of the colon for each group. Scale bar = 100 µm. LIPUS alleviated (**B**) histological damage, (**C**) muscle thickness, and (**D**) villi shortening in the colon for each group. Key: *, ^#^, and ^†^ denote significant differences from the Sham, LPS, and LPS+LIPUS 0.5 groups, respectively; *, *p* < 0.05; **, ^##^, ^††^, *p* < 0.01; ***, ^###^, ^†††^, *p* < 0.001 (*n* = 5).

Histopathological examinations were performed using H&E-stained sections to characterize colonic injury. Representative colonic images were captured at 100× magnification for each group (Figure 2A). Histological scores showed significantly reduced LPS-induced colonic damage with decreased crypt destruction and partial epithelial barrier preservation in the LPS+LIPUS 0.5 and LPS+LIPUS 1.0 groups compared to the LPS group (7.80 ± 4.09, 4.20 ± 0.45 vs. 16.00 ± 5.10, both *p* < 0.01; Figure 2A,B). The LPS+LIPUS 1.0 group showed a significant reversal in muscle thickening compared to the LPS group (22.78 ± 3.00 vs. 42.10 ± 7.23, *p* < 0.001; Figure 2C). However, muscle thickness did not differ significantly between the LPS+LIPUS 0.5 and LPS groups. Furthermore, there was marked villus shortening in the LPS group (Figure 2D). LIPUS significantly reduced LPS-induced mucosal damage, ameliorating villi length in the LPS+LIPUS 1.0 group compared to the LPS group. These results suggested that LIPUS attenuates LPS-induced gut barrier dysfunction in mice.

### 2.3. LIPUS Ameliorates LPS-Induced Lesions in the Brain

**Figure 3:**
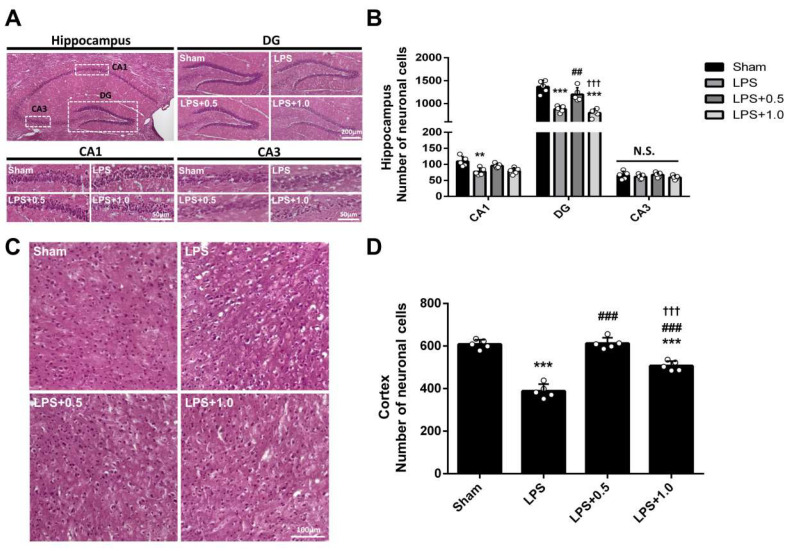
Effects of LIPUS on histopathological changes in the brain of LPS-treated mice. (**A**) Representative images of H&E staining in the hippocampus. DG: Scale bar = 200 µm. CA1 and CA3: Scale bar = 50 µm. (**B**) The number of neurons in hippocampal CA1, DG, and CA3 regions for each group. (**C**) Representative images of H&E staining in the cortex. Scale bar = 100 µm. (**D**) The number of neurons in the cortex for each group. Key: *, ^#^, and ^†^ denote significant differences from the Sham, LPS, and LPS+LIPUS 0.5 groups, respectively; **, ^##^, *p* < 0.01; ***, ^###^, ^†††^, *p* < 0.001 (*n* = 5). N.S.: not significant.

H&E staining showed histopathological changes in the hippocampus and cortex (Figure 3A,C). The neurons in the hippocampus and cortex were arranged neatly and tightly, with clear nuclei in the Sham group. In contrast, the neuronal arrangement was disordered, the intercellular space was increased, and many necrotic and apoptotic cells were observed in the LPS-treated groups. Mouse brains in the LPS+ LIPUS 0.5 group showed marked improvements in the pyramidal neurons, which were similar to the normal structures in the Sham group. LIPUS treatment significantly attenuated the LPS-mediated reduction in neuron numbers in hippocampal DG regions in the LPS+LIPUS 0.5 group (881.20 ± 69.00 vs. 1198.60 ± 149.61, *p* < 0.01; Figure 3B). Like the hippocampus results, LIPUS treatment significantly attenuated the LPS-mediated reduction in neuron numbers in the cortex in the LPS+LIPUS 0.5 group (388.40 ± 32.56 vs. 612.60 ± 26.29, *p* < 0.05; Figure 3D). However, LIPUS treatment did not attenuate the LPS-mediated reduction in neuron numbers in the hippocampus in the LPS+LIPUS 1.0 group (Figure 3B).

### 2.4. LIPUS Ameliorates LPS-Induced Microglial Activation and Neuronal Cell Death

**Figure 4:**
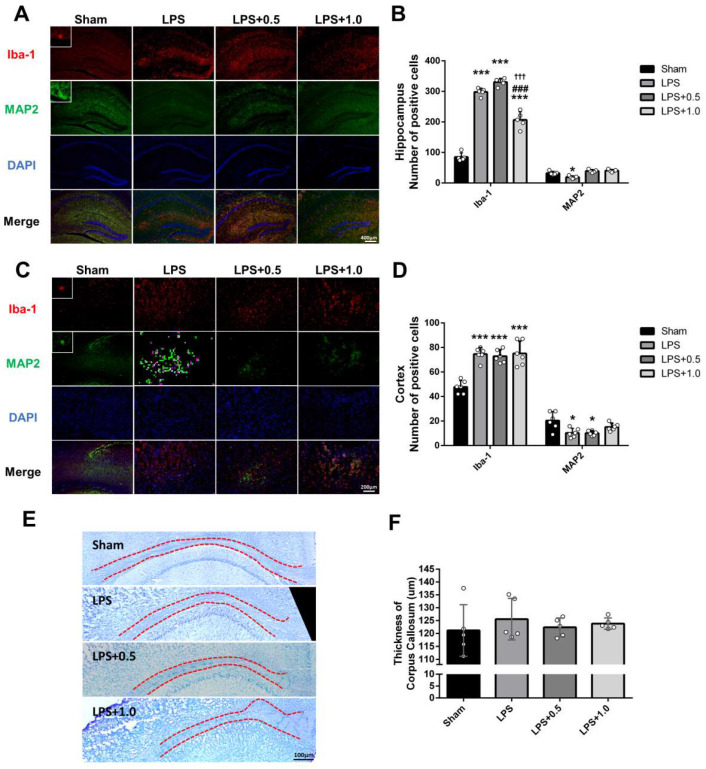
Iba-1 and MAP2 expression in the inflamed brain of LPS-induced mice. (**A**) Representative immunofluorescent staining in the hippocampus for Iba-1 (red) and MAP2 (green). Scale bar = 400 μm. (**B**) Quantification of Iba-1 and MAP2 cell numbers in the hippocampus for each group. (**C**) Representative immunofluorescent staining in the cortex for Iba-1 (red) and MAP2 (green). Scale bar = 200 µm. (**D**) Quantification of Iba-1 and MAP2 cell numbers in the cortex for each group. (**E**) Representative images of LFB staining in the corpus callosum for four groups. (**F**) Bar graph of the mean thickness of the corpus callosum. Key: *, ^#^, and ^†^ denote significant differences from the Sham, LPS, and LPS+LIPUS 0.5 groups, respectively; *, *p* < 0.05; ***, ^###^, ^†††^, *p* < 0.001 (*n* = 5).

Iba-1 was used as a marker of microglial activation induced by inflammation [27]. MAP2 is a marker for mature dendrites. It is likely that a degree of MAP2 loss is attributable to the neuronal death. The hippocampus and cortex were immunofluorescently stained with antibodies against Iba-1 and MAP2 to examine whether the LIPUS treatment alleviated LPS-induced microglial activation and neuronal cell loss (Figure 4A,C). The numbers of Iba-1-positive cells in the hippocampus and cortex were significantly higher in the LPS group than in the Sham group (297.33 ± 11.24 vs. 83.30 ± 12.00, 74.67 ± 5.28 vs 47.80 ± 5.23, both *p* < 0.001; Figure 4B,D). LIPUS treatment significantly attenuated LPS-induced hippocampal Iba-1 activity in the LPS+LIPUS 1.0 group (297.33 ± 11.24 vs. 204.7 ± 24.35, *p* < 0.001; Figure 4B) but not in the LPS+LIPUS 0.5 group. However, LIPUS did not block the LPS-induced Iba-1 activity in the cortex (Figure 4D). Furthermore, LPS caused a significant decrease in MAP2-positive neuronal cell loss in the hippocampus and cortex. While not significant, LIPUS treatment afforded partial protection against LPS-mediated neuronal loss in the hippocampus and cortex of mice in the LPS+LIPUS 1.0 group to that of the Sham group. In addition, no significant differences were found in the thickness of the corpus callosum among the four groups (Figure 4E,F).

### 2.5. LIPUS Ameliorates LPS-Induced Apoptosis in the Brain

**Figure 5:**
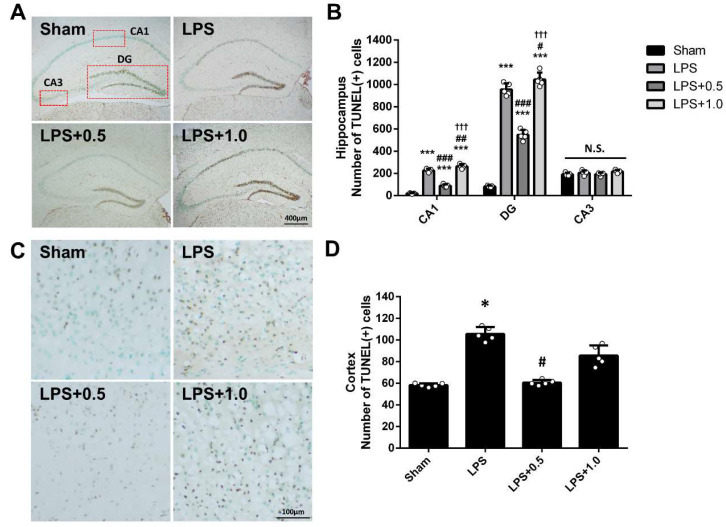
Effects of LIPUS on the apoptotic cells in the brain of LPS-treated mice. (**A**) Representative images of TUNEL staining in the hippocampus. Scale bar = 400 µm. (**B**) The number of TUNEL-positive cells in hippocampal CA1, DG, and CA3 regions for each group. (**C**) Representative images of TUNEL staining in the cortex. Scale bar = 200 µm. (**D**) The number of TUNEL-positive cells in the cortex for each group. Key: *, ^#^, and ^†^ denote significant differences from the Sham, LPS, and LPS+LIPUS 0.5 group, respectively; *, ^#^, *p* < 0.05; ^##^, *p* < 0.01; ***, ^###^, ^†††^, *p* < 0.001 (*n* = 5). N.S.: not significant.

TUNEL-positive cells were quantified in the hippocampus and cortex to examine whether LIPUS treatment protects neurons from LPS-induced apoptosis (Figure 5A,C). Consistent with the results of Figure 3, many apoptotic cells were observed in the LPS-treated groups. LIPUS treatment significantly reduced apoptotic cells in the hippocampal CA1 and DG regions and the cortex in the LPS+LIPUS 0.5 group (222.67 ± 15.90 vs. 84.50 ± 16.48, 937.17 ± 64.83 vs. 525.00 ± 66.30, 105.55 ± 6.37 vs. 60.60 ± 2.43, all *p* < 0.05; Figure 5B,D). However, LIPUS treatment did not reduce neuronal apoptosis in the LPS+LIPUS 1.0 group. These results suggest a neuroprotective effect of LIPUS treatment on LPS-induced neuroinflammation at the lower intensity of 0.5 W/cm^2^.

## 3. Discussion

Neuroinflammation has been reported to contribute to the development of neurodegenerative diseases and is considered an important treatment target. LPS-induced animal models are widely used to investigate systemic inflammation, showing harmful effects on the gut and brain [28]. In this study, quantitative analysis showed that LIPUS significantly attenuated microglial activation, neuronal cell loss, and apoptosis in the brain. We showed that abdominal LIPUS stimulation could alleviate brain injuries by improving colonic damage, suggesting that the gut–brain axis (GBA) may be involved in this process.

The cholinergic anti-inflammatory pathway has been extensively investigated due to its modulation of the immune response [29]. The neural innervation within the spleen is thought to influence systemic inflammation via the cholinergic anti-inflammatory pathway. Vagus nerve stimulation has been shown to treat arthritis in animal models, and there is a direct link between the inflammatory responses and the cholinergic nervous system [30]. The neural reflexes in inflammation are triggered while the vagus nerve is stimulated with electrical current and subsequently attenuated the inflammation in the injured tissue. Another recent work demonstrated that inflammation and tissue damage can be alleviated in the renal ischemic reperfusion injury of mice by abdominal LIPUS stimulation [31]. These techniques have revealed anti-inflammatory effects for chronic inflammatory diseases via physical stimulation. In the emerging technique of noninvasive neuromodulation, increasing evidence showed the possibility of using such methods to treat disease by direct or indirect stimulation. Previous study showed that LIPUS stimulation of the spleen treat inflammatory arthritis [32]. Recently, one study showed LIPUS stimulation within organs provides a new tool for site-specific neuromodulation for modulate physiological functions [33]. One advantage of LIPUS for clinical application is that it can noninvasively target organs within deep body place. Moreover, LIPUS stimulation could potentially minimize side effects and enhance treatment for various inflammatory conditions.

The gut–brain axis showed bidirectional communication between the enteric and CNS that not only maintains the gastrointestinal homeostasis but also has various effects on emotional and cognitive functions of the brain [34]. The mechanisms of gut–brain communication involve multiple pathways, which include the vagus nerve, chemicals produced by gut microbes, inflammation, and neurotransmitters. LPSs enter the circulation and trigger systemic inflammatory responses. Peripheral inflammation affects the brain through cytokines in the systemic circulation that access the brain [35]. The blood–brain barrier (BBB) is formed by endothelial cell tight junctions in brain capillaries that protect against circulating toxins. Breakdown of the BBB occurs in many brain diseases and is often induced by inflammatory mechanisms. Proinflammatory mediators in the circulation can reach the brain while the BBB is disrupted [36]. It has been shown that endotoxin produced by the gut results in the neuroinflammation under conditions of the BBB disruption in a colitis animal model [37]. Proinflammatory cytokines in the brain can lead to neuronal death and brain dysfunction, and neuroinflammation is now considered a key factor in many neurodegenerative diseases [38]. The exact mechanisms underlying LIPUS modulation of the anti-inflammatory pathway via the gut–brain axis is unknown. Numerous studies have demonstrated that LIPUS can stimulate peripheral nerves, partially via a mechanical effect that regulates mechanosensitive ion channels on neural cell membranes or induce cell membrane porosity [39,40]. Here, our data suggest that LIPUS may alleviate the neuronal damage and microglial activation through attenuation of gut barrier disruption.

Intestinal inflammation increases the number of smooth muscle cells, leading to the thickening of the smooth muscle layer [41]. In addition, the spleen is the largest peripheral lymphatic organ and plays a key role in endotoxin-induced immune responses. IBD is associated with colonic muscle thickening, colonic villi shortening, and spleen enlargement [42,43]. In this study, LPS treatment caused splenomegaly, and abdominal LIPUS did not affect spleen weight in LPS-treated mice at both 0.5 and 1.0 W/cm^2^ intensities (Figure 1). Therefore, LIPUS stimulation did not ameliorate endotoxin-induced spleen edema, suggesting LIPUS did not suppress general immune responses. As in our previous study, the LIPUS stimulation could modulate the BBB permeability and reduced neuronal death and apoptosis in the injured brain [44]. Here, LIPUS stimulation could alleviate the structure damage of gut. This was probably due to the fact that LIPUS has beneficial effects on the gut barrier. This phenomenon requires more study to explain its mechanisms and to optimize the parameters of LIPUS.

The intestinal barrier is one of the most crucial defense systems for maintaining homeostasis in the body [45]. Intestinal barrier dysfunction facilitates the entry of endotoxic substances into the circulation, inducing systemic inflammation and neuroinflammation. Applying LIPUS to the abdomen has been shown to attenuate the severity of gut inflammation by improving colonic histological damage [26]. To identify the influences of abdominal LIPUS stimulation in the colon of LPS-treated mice, we examined the colon histology of LPS-treated mice using H&E staining (Figure 2). Consistent with a previous study, abdominal LIPUS improved muscle thickness and villi length in the colon of LPS-treated mice. In future experiments, we intend to investigate whether gut microbiome structure is altered following abdominal LIPUS stimulation. We will also explore the beneficial effects of abdominal LIPUS on reducing gut barrier permeability.

Hippocampus and cortex structural changes are implicated in brain injury (Figure 3). Based on the observation in H&E staining, LIPUS attenuated LPS-induced microstructural lesions, indicated by decreased necrotic neurons and increased healthy neurons in the CA1 and DG regions. These findings suggested that LIPUS stimulation reversed pathology associated with LPS-induced damage. Furthermore, previous reports have shown that apoptosis is involved in LPS-induced neuroinflammation [46,47]. Our results showed that LIPUS alleviated neuronal apoptosis in the hippocampus and cortex of the LPS+LIPUS 0.5 group (Figure 5), which may be a neuroprotective effect of LIPUS against LPS-induced brain damage at the lower intensity of 0.5 W/cm^2^. It has been reported that microglial activation contributes to neuroinflammation progression [48]. The number of microglia (Iba-1-positive cells) was higher, while the number of neurons (MAP2-positive cells) was lower in the LPS-treated groups than in the Sham group. Abdominal LIPUS stimulation alleviated microglial activation and neuronal cell loss in the hippocampus of the LPS+LIPUS 1.0 group compared to the LPS group. However, abdominal LIPUS did not affect the microglial activation and neuronal cell loss in the LPS+LIPUS 0.5 group. Therefore, the neuroprotective effects of abdominal LIPUS on different brain regions depend on the ultrasound parameters. Selecting the appropriate LIPUS parameters is challenging. Further investigations are required to determine the optimal ultrasound parameters for various brain regions. Based on our results, LIPUS may be a potential tool for alleviating neuroinflammation-related brain injuries.

Compared to chemotherapeutics, LIPUS stimulation is a safe and non-invasive form of neuromodulation, which is broadly applied in various diseases. An ultrasound stimulus is determined by several parameters: operating frequency, intensity, duration, and pulse repetition frequency. Each of these parameters may have different effects on the experimental outcome. In this study, one set of parameters was selected based on our previous work. Further investigations of optimal ultrasound parameters are needed. Recently, it has been revealed that LIPUS exerts a significant therapeutic effect in animal models of stroke, Alzheimer’s disease, and Parkinson’s disease [24,49,50]. Although the findings presented in our data were consistent with previous reports, there are some limitations to this study. The first limitation was the use of a single-element transducer made it difficult to provide targeted sonication. A phased-array focused transducer could be designed to achieve more localized sonication in the target organs or regions. Further experiments will be needed to explore the feasibility of precision LIPUS neuromodulation by a phased-array focused ultrasound. Next, development of an integrated LIPUS transducer that can combine focused ultrasound and image to the localized site could enable more accurate targeting. In addition, the aim of this study was to investigate the possible effects in the neuroinflammatory responses after abdominal LIPUS stimulation. Behavioral or possible pathways are needed to investigate in future works. Another limitation is that this systemic model is not a colitis model as the DSS colitis model, but it can mimic a bacterial infection by inducing neuroinflammation, which contributes to the sickness response. In this study, an unfocused ultrasound was used for stimulation. Thus, the third limitation is that the real mechanisms of LIPUS treatment are difficult to clarify because the LIPUS exposure was applied to the whole abdomen of the mice. Our group is currently establishing the image-guided, focused LIPUS to stimulate the specific organ or regions for further studies.

## 4. Materials and Methods

### 4.1. Ultrasound Setup and Treatments

**Figure 6:**
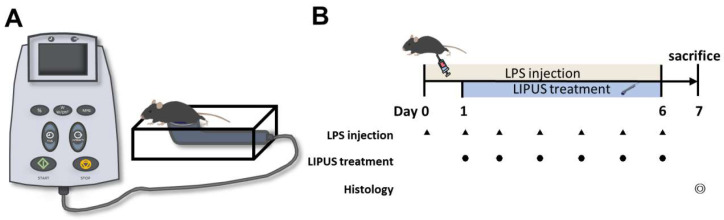
The experimental system and flow diagram. (**A**) Schematic diagram of the ultrasound system setup and treatment with transabdominal stimulation. (**B**) The study’s time course. Mice were treated daily with LIPUS for six days and sacrificed on day 7 after the LPS injection. ▲: LPS injection. ●: LIPUS treatment.

An ultrasound generator (ME740; Mettler Electronics, Anaheim, CA, USA) and a 1 MHz plane transducer with a 4.4 cm^2^ effective radiating area (ME7413; Mettler Electronics) were used to generate LIPUS with 2-ms burst lengths at a 20% duty cycle and a repetition frequency of 100 Hz. The average spatial intensity over the plane transducer head was 0.5 or 1.0 W/cm^2^, measured with a radiation force balance (Precision Acoustics, Dorset, UK) in degassed water. Before LIPUS treatment, the mouse’s abdomen was shaved. The area between the transducer and the abdomen was covered with ultrasound transmission gel (Pharmaceutical Innovations, Newark, NJ, USA) to maximize the transmission of the ultrasound. The entire abdomen between the diaphragm and groin was treated (Figure 6A). In order to reduce the thermal effect of ultrasound, each application lasted 5 min, with a 5 min interval between the first, second, and third applications. The total LIPUS stimulation time was 15 min daily. Different combinations of parameters result in various modulatory effects. The parameters of the LIPUS stimulation were chose based on the results of our previous experiments [22,51].

### 4.2. Animal Model

Male C57BL/6J mice weighing 22–25 g were purchased from LASCO. Animals were maintained under a 12/12 h light/dark cycle with free access to food and water. All procedures involving animals were conducted according to the guidelines for the Care and Use of Laboratory Animals published by the United States National Institutes of Health. This study protocol was approved by the Animal Care and Use Committee of National Yang Ming Chiao Tung University (1100224, 5 February 2021).

LPS (Sigma-Aldrich, St. Louis, MO, USA) was dissolved in saline. The chosen LPS dose (0.75 mg/kg) did not induce mortality in mice but induced systemic inflammation. Animals were divided into four treatment groups: Sham, LPS, LPS+LIPUS 0.5, and LPS+LIPUS 1.0. Mice in the Sham and LPS groups were intraperitoneally injected with vehicle (saline) or LPS, respectively, daily for seven days (Figure 6B). LIPUS was applied to mice in the LPS+LIPUS 0.5 and LPS+LIPUS 1.0 groups for six consecutive days at ultrasound intensities of 0.5 and 1.0 W/cm^2^, respectively. Mice in these two groups were anesthetized with isoflurane mixed with oxygen before LIPUS was applied. Mice in the Sham and LPS groups were also anesthetized with isoflurane mixed with oxygen for this period.

### 4.3. Tissue Processing

Mice were deeply anesthetized and perfused intracardially with phosphate-buffered saline (PBS) followed by 4% paraformaldehyde. Their brain, colon, and spleen were collected, post-fixed in 4% paraformaldehyde overnight, and transferred to PBS containing 30% sucrose for cryoprotection. Serial coronal sections (10 μm) were made using a microtome. Three brain or colon sections were imaged for five mice per group for analysis. The spleen was surgically removed and cleaned of the extraneous tissue. It was rinsed with cold isotonic saline, dried by blotting between two pieces of filter paper and weighed on an electronic balance to estimate spleen edema.

### 4.4. Histopathology

For each case, the three most representative hematoxylin and eosin (H&E)-stained colon and brain tissue sections were collected for histological evaluation on day 7. These sections were analyzed by light microscopy (Nikon E100, Nikon corporation, Tokyo, Japan) at 100× magnification. The colon was scored as previously described [52], including its inflammation (0–3), extent (0–3), regeneration (0–4), crypt damage (0–4), and percent involvement (0–4). Detailed scores are shown in Table 1. Each section was scored for each feature separately by calculating the product of the grade for that feature and the percentage involvement. The brain slices were H&E-stained to visualize the general cellular structure in the hippocampus and cortex. The number of neurons in the cortex and hippocampal cornu Ammonis subfields 1 (CA1) and 3 (CA3) and dentate gyrus (DG) was counted with the ImageJ software version 1.54d (National Institute of Health, Bethesda, MD, USA). Furthermore, a DNA fragmentation detection kit detected apoptotic cells characterized by DNA fragmentation. Methyl green staining was used for the morphological assessment and characterization of normal and apoptotic cells. Cells with the nuclear-staining characteristic of terminal deoxynucleotidyl transferase-mediated dUTP nick end labeling assay-positive (TUNEL^+^) cells were counted in six mice per group. In this study, we quantified the number of cells that were positive for H&E and TUNEL staining in a 420 × 420 μm^2^ area and the number of cells positive for Iba-1 and MAP2 in a larger area of 1400 × 870 μm^2^, both in three non-overlapping fields of the cortex. To obtain the mean thickness, the corpus callosum from five slices, each with a thickness of 10 µm and spaced 300 µm apart, were stained with Luxol fast blue staining (LFB) to visualize the tissue structure. The stained slices were imaged at 10× magnification using a bright field microscope (Olympus BX-63, Tokyo, Japan), and four mice per group were included in the analysis. The corpus callosum thickness was measured by delineating the region of interest (1180 × 327 µm^2^) using ImageJ software. The stereotaxic coordinates for the studied area were determined relative to bregma as follows: anteroposterior, −3.5 mm; mediolateral, 2.5 ± 1 mm; dorsoventral, 1.3 ± 0.5 mm.

### 4.5. Immunofluorescence

Brain tissues were processed for immunofluorescent detection. The primary antibodies used were anti-ionized calcium-binding adaptor molecule-1 (Iba-1; 1:250; GTX100042, GeneTex, Alton Pkwy Irvine, CA, USA) and anti-MAP2 (1:200; GTX11267, GeneTex, Alton Pkwy Irvine, CA, USA). The brain sections were incubated with primary antibodies overnight, then washed and incubated with either Alexa Fluro 488- (1:500; GTX213111, GeneTex, Alton Pkwy Irvine, CA, USA) or Alexa Fluro 594- (1:500; GTX213110, GeneTex, Alton Pkwy Irvine, CA, USA) tagged secondary antibodies at room temperature. The number of cells positive for Iba-1 and MAP2 was counted in three non-overlapping fields in the cortex and hippocampus at ×200 magnification. Next, the sections were incubated in a 1:200 dilution of a TRITC-conjugated goat anti-rabbit secondary antibody and FITC-conjugated goat anti-mouse secondary antibody for 1 h at room temperature and then washed three times with PBS for 10 min. Then, the sections were stained with DAPI solution for 10 min at room temperature. Finally, fluorescence images were obtained by fluorescence microscopy (Leica DM 6000B; Mannheim, Germany), and the number of cells was counted with the ImageJ software.

### 4.6. Data Analysis

All data are presented as means ± standard deviations. One-way analyses of variance with a Bonferroni post hoc test were used to compare histological data between groups. All results with a p-value of ≤0.05 were considered statistically significant.

## 5. Conclusions

In summary, transabdominal LIPUS stimulation reduced microglial activation, neuronal cell loss, and apoptosis in the brain of LPS-treated mice by protecting the gut barrier. Our data showed that transabdominal ultrasound might be a novel strategy for improving systemic inflammation pathological processes via the related gut–brain axis pathway. These findings suggest that LIPUS could be a potential therapeutic approach for treating IBD and neuroinflammation-induced brain diseases.

## Figures and Tables

**Table 1:** Histological grading of colitis.

Feature Graded	Grade	Description
Inflammation	0	None
	1	Slight
	2	Moderate
	3	Severe
Extent	0	None
	1	Mucosa
	2	Mucosa and submucosa
	3	Transmural
Regeneration	4	No tissue repair
	3	Surface epithelium not intact
	2	Regeneration with crypt depletion
	1	Almost complete regeneration
	0	Complete regeneration or normal tissue
Crypt damage	0	None
	1	Basal 1/3 damaged
	2	Basal 2/3 damaged
	3	Only surface epithelium lost
	4	Entire crypt and epithelium lost
Percent involvement	1	1–25%
	2	26–50%
	3	51–75%
	4	76–100%

## Data Availability

The data that support the findings of this study are available within the article and from the corresponding authors upon reasonable request.
